# Coping with levels of explanation in the behavioral sciences

**DOI:** 10.3389/fpsyg.2015.00213

**Published:** 2015-02-25

**Authors:** Giuseppe Boccignone, Roberto Cordeschi

**Affiliations:** ^1^Department of Computer Science, University of MilanMilan, Italy; ^2^Department of Philosophy, Sapienza University of RomeRome, Italy

**Keywords:** human behavior, levels of explanation, neuroscientific models, functional models, computational models, cognitive architectures, mechanisms, reductionism

## Introduction

This Research Topic aimed at deepening our understanding of the levels and explanations that are of interest for cognitive scientists, neuroscientists, psychologists, behavioral scientists, and philosophers of science.

Indeed, contemporary developments in neuroscience and psychology suggest that scientists are likely to deal with a multiplicity of levels, where each of the different levels entails laws of behavior appropriate to that level (Berntson et al., 2012). Also, gathering and modeling data at the different levels of analysis is not sufficient: the integration of information across levels of analysis is a crucial issue.

Given such state of affairs, a number of interesting questions arise. How can the autonomy of explanatory levels be properly understood in behavioral explanation? Is reductionism a satisfactory strategy? How can high-level and low-level models be constrained in order to be actually explanatory of both behavioral and neurological or molecular evidence? What is the kind of relationship between those models?

## Plurality of levels with and beyond marr

Marr (1982) distinguished between three levels of explanation, the *what/why* level (computational theory), the *how* level (algorithm), and the *physical realization* level (implementation). His influential framework has had a far-reaching influence in both neuroscience and cognitive science over the years and it has become a sort of paradigm. However, the tremendous developments in such sciences suggest that there will hardly be only three levels of explanation.

For instance, Castelfranchi (2014) claims for several different layers of “theory”: the cognitive representations and mechanisms; the neural processes; the evolutionary history and adaptive functions of our cognition and behaviors; the social structures and dynamics with their relation and feedbacks on individual minds and behaviors; the historical and cultural mechanisms; the developmental paths.

Clearly, on the one hand, dealing with such complexities calls for models that simulate those processes so that they can be used as explanatory tools, i.e., instances of the “synthetic” method (Cordeschi, 2002). In this perspective, Conte and Paolucci (2014) make the point that simple recipes have prevailed up to now and shadowed the application of rich cognitive models. As a viable solution, they discuss Agent Based Modeling and its role at the highest behavioral level of Computational Social Science.

On the other hand, to cope with multi-level complexity, Abney et al. (2014) propose explanatory pluralism. They present one concrete example, the analysis of a corpus of conversing individuals solving a joint decision-making task, performed by using decision-making at the behavioral level, confidence sharing at the linguistic level, acoustic energy at the physical level.

A further interesting issue is that of the objective vs. subjective meaning of the explanatory levels. Varma (2014) discusses how Marr's approach focused on the objective meaning of each level—how it supports computational models that correspond to cognitive phenomena—and he develops a complementary analysis of the subjective meaning of each level—how it helps cognitive scientists understand cognition. With the goal of showing that different kinds of explanation arise because we have different kinds of explanatory concerns, a clear case study is proposed by Wilkinson (2014) by using contrasting theories of delusional misidentification.

## Relationships, constraints and mechanisms

Addressing any level of description involves a certain degree of realist commitment at that level, which, in turn, has some consequences on the problems of reduction and of causality between levels. In this respect, one important case study is presented by Albertazzi and Poli (2014), who address the conundrum of color. They claim that color is a different entity for each level of reality and it generates different observables in the epistemologies of the different sciences.

Ramos (2014) introduces the hypothesis that the sophisticated psychological constructs classically associated with the concept of mental representation are essentially of the same nature of simple interactive behaviors. Thus, the capacity of generating elaborated mental phenomena like beliefs and desires emerges gradually during evolution, and social interaction. Here, mental representations are biological phenomena whose construction is achieved by a correlational mechanism of information exchange with the external world.

In a related perspective and in order to cope with the multiscale nature (Abney et al., 2014) of cognitive and behavioral phenomena, Costa and Ferraro (2014) argue that a statistical mechanics approach is almost inescapable. Starting from very simple systems, connectivity gives rise to levels of increasing functional complexity.

Here the key issue is that, at any level, systems obey laws holding for the lower levels; meanwhile, they are subjected to new constraints (related to and implemented through neural structures). These, in turn, generate new features, like novel patterns of activity, requiring adequate levels of representation in terms of model structures and variables.

Indeed, accounting for constraints is a central point: as Abney et al. (2014) put it, “mapping across levels should create mutual constraints, in that levels should be consistent, if qualitatively, with each other.” A hallmark of the present state of research in cognitive/behavioral sciences is that one is generally ignorant of how exactly to cast the different levels into a grounded relationship. In this case the notions of structure and architecture—and related graphical modeling tools—become crucial, since they are necessary to embody constraints at the chosen level of explanation (Boccignone and Cordeschi, 2007, 2012).

The exploitation of structure/architecture as a tool for bridging intra- and inter-level constraints has the merit of paving the way for reconciling rational or information-based analyzes (Danks, 2008) with mechanism-based explanations (Bechtel and Abrahamsen, 2005). As fostered by Castelfranchi (2014), “laws” are not enough, both the “why” and “how” must be addressed.

In this respect, Datteri and Laudisa (2014) lucidly address the subtelties of graphical explanations, making the case for the relationship between box-and-arrow (BA) explanations and neuroscientific mechanism descriptions (NMDs). The interesting point raised by Datteri and Laudisa is that the BA analysis imposes constraints on the formulation of the NMD by postulating a number of regularities to be sought for in the neural activities of the system. Conversely, the NMD constrains the space of the possible BA analyzes of the system by postulating a number of neural regularities.

## Conclusion

Taken together, the papers in “What levels of explanation in the behavioral sciences” give us some important indications of where the field is going and also demonstrate how lively and open the field is today.

We hope this Research Topic paves the way to new avenues and challenges for future work.

### Conflict of interest statement

The authors declare that the research was conducted in the absence of any commercial or financial relationships that could be construed as a potential conflict of interest.
